# Human fibronectin extra domain B as a biomarker for targeted therapy in cancer

**DOI:** 10.1002/1878-0261.12705

**Published:** 2020-06-15

**Authors:** Relinde I. Y. Lieverse, Damiënne Marcus, Alexander M. A. van der Wiel, Evert J. Van Limbergen, Jan Theys, Ala Yaromina, Philippe Lambin, Ludwig J. Dubois

**Affiliations:** ^1^ The M‐Lab Department of Precision Medicine GROW – School for Oncology and Developmental Biology Maastricht University The Netherlands; ^2^ Department of Radiation Oncology (MAASTRO) GROW – School for Oncology and Developmental Biology Maastricht University Medical Centre The Netherlands

**Keywords:** angiogenesis, biomarker, cancer, extra domain B, immunotherapy, targeted therapy

## Abstract

The extracellular matrix protein fibronectin contains a domain that is rarely found in healthy adults and is almost exclusively expressed by newly formed blood vessels in tumours, particularly in solid tumours, different types of lymphoma and some leukaemias. This domain, called the extra domain B (ED‐B), thus has broad therapeutic potential. The antibody L19 has been developed to specifically target ED‐B and has shown therapeutic potential when combined with cytokines, such as IL‐2. In this review article, we discuss the preclinical research and clinical trials that highlight the potential of ED‐B targeting for the imaging and treatment of various types of cancer. ED‐B‐centred studies also highlight how proper patient stratification is of utmost importance for the successful implementation of novel antibody‐based targeted therapies.

## Graphical abstract

The extracellular matrix protein fibronectin contains a domain that is rarely found in healthy adults and is almost exclusively expressed by newly formed blood vessels in tumours, the extra domain B (ED‐B). This review summarizes preclinical research and clinical trials on the potential of antibody‐based ED‐B targeting for tumour visualization and treatment.

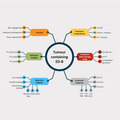

**Abbreviations**
(scFv)2: dimeric scFv
aPD(L)1: anti‐programmed cell death protein (ligand) 1
cFN: cellular fibronectin
ECM: extracellular matrix, ED‐A, extra domain A
ED‐A: extra domain A
ED‐B: extra domain B
ELISA: enzyme‐linked immunosorbent assay
FN: fibronectin
GM‐CSF: granulocyte–macrophage colony‐stimulating factor
IgG: immunoglobulin G
IITs: intralesional immunotherapies
INF‐γ: interferon gamma
MHCI: major histocompatibility complex I
Mio IU: million international unit
NG: not given
NSCLC: non‐small‐cell lung cancer
PET: positron emission tomography
pFN: plasma fibronectin
qPCR: quantitative polymerase chain reaction
RIT: radioimmunotherapy
SABR: stereotactic ablative body radiotherapy
SIP: small immune protein
SPECT: single‐photon emission computed tomography
TBMR: tumour to bone marrow ratio
TBR: tumour‐to‐blood ratio
TNF‐α: tumour necrosis factor alpha
UN: unknown
VEGF: vascular endothelial growth factor

## 1 Introduction

Cancer is estimated to have caused 10.5 million deaths worldwide in 2020, mostly due to metastatic cancer (IARC, 2020). The main therapeutic approaches used to treat metastatic cancer include surgery, chemotherapy and radiotherapy, singly or in combination. Chemotherapy is the treatment that is most commonly used (ACS, 2019), but it often has a limited impact on cancer survival (Morgan *et al*., 2004). Chemotherapeutics lack selectivity for tumour cells over healthy cells, resulting in fast‐reaching systemic toxicity. Targeted therapy reduces toxicity for healthy tissues due to its specific delivery of, for example, cytokines, cytotoxic agents, chemotherapy drugs and radioisotopes, to the genes, proteins, cancer cells or sites that promote cancer growth (Padma, 2015).

Amongst potential targets, fibronectin (FN) that contains a domain, called the extra domain B (ED‐B), is a promising candidate. It was discovered by Borsi *et al*. (1985) and is almost exclusively expressed by newly formed blood vessels in tumours, and thus has broad therapeutic potential (Borsi *et al*., 1987; Zardi *et al*., 1987). FN that does not contain the ED‐B plays an important role in various biological processes, including thrombosis, haemostasis, maintenance of normal cell morphology, cell adhesion, migration, wound healing and oncogenic transformation (Ebbinghaus, 2004). In contrast, ED‐B‐containing FN is rarely found in healthy adults and is associated with heart and vasculature processes, including embryonic development, tissue regeneration and chronic‐active pathological conditions associated with new blood vessel formation, including cancer (Menrad and Menssen, 2005; Schiefner *et al*., 2012).

Fibronectin is an extracellular matrix (ECM) protein that binds to integrins and to other ECM proteins (Wierzbicka‐Patynowski and Schwarzbauer, 2003). The alternative splicing of the primary FN transcript results in the expression of three different domains (V or IIICS, ED‐A or EIIIA, and ED‐B or EIIIB), singly or in combination. This alternative splicing of FN might explain why for ED‐B it contains 91‐amino acid, created by a type III homology repeat (Kumra and Reinhardt, 2016; Schiefner *et al*., 2012). In cancer and transformed cells, the alternative splicing of FN is increased, leading to an increased expression of ~ 20 different FN isoforms that contain one or more domains (Ebbinghaus *et al*., 2004; Kumra and Reinhardt, 2016; Schiefner *et al*., 2012). Furthermore, each FN domain interacts with different circulating proteins in the blood, such as heparin/heparan sulfate moieties, ECM protein and cell‐surface integrin receptors (Kumra and Reinhardt, 2016; Menrad and Menssen, 2005). Although the role of the ED‐B domain is not entirely clear, it has been shown to support the processes of tube formation, cell adhesion, endothelial cell proliferation, correct FN matrix assembly, neovascularization and the increased expression of vascular endothelial growth factor (VEGF) during angiogenesis (Kumra and Reinhardt, 2016; To and Midwood, 2011).

In this review, we provide an overview of the latest, clinically related information regarding the specific expression of ED‐B in the cancer microenvironment, including its functions, how to detect and to exploit EB‐B for imaging and therapeutic purposes, respectively, especially when combined with conventional treatment possibilities such as radiotherapy.

## 2 The functions of ED‐B

In physiological conditions, angiogenesis is activated in a transient and localized manner during, for example, wound healing and inflammation. At the start of angiogenesis, endothelial cells secrete proteases at a specific location to degrade the ECM, allowing endothelial cells to migrate from blood vessels and into the perivascular space, where they proliferate to start the formation of new blood vessels (Ebbinghaus *et al*., 2004). This tightly controlled process is regulated by an equilibrium being achieved between stimulatory and inhibitory angiogenic factors (Haibe *et al*., 2020).

In all cancers, however, this equilibrium is disturbed, resulting in poorly constructed and uncontrolled angiogenesis occurring within the tumour (Haibe *et al*., 2020). The tumour vasculature often contains high levels of ED‐B, and shows increased permeability and a higher interstitial fluid pressure compared with normal blood vessels, thus forming a major barrier to the uptake of therapeutic agents (Menrad and Menssen, 2005). Because ED‐B is specifically present in the tumour microenvironment, its targeting presents a promising therapeutic option for the delivery of therapeutic agents, such as cytokines, chemotherapeutics, cytotoxic agents and radioisotopes, to tumours (Kumra and Reinhardt, 2016).

Two forms of FN exist: soluble FN present in plasma (pFN) and cellular FN (cFN), which is present in the ECM. pFN is synthesized and secreted without ED‐A or ED‐B segments by hepatocytes in the liver as double polypeptide chains, which are joined together by disulfide bridges at their C termini (Schiefner *et al*., 2012; To and Midwood, 2011). Its concentration in plasma is high (max ~ 0.65 mg·mL^−1^), which makes it easy to detect. By contrast, cFN is synthesized and secreted with ED‐A and/or ED‐B segments by various cell types, including endothelial cells, macrophages, smooth muscle cells and fibroblasts as a disulfide‐linked, tail‐to‐tail dimer (Schiefner *et al*., 2012; To and Midwood, 2011), but its concentration in plasma is negligible (Kumra and Reinhardt, 2016; Prakash *et al*., 2015; Pujuguet *et al*., 1996). cFN, however, is easily detected in urine using indirect ELISA (Arnold *et al*., 2016). ED‐B has a 3D structure composed of 2 antiparallel β sheets with 25 amino acid side chains, consisting of 3 and 4 β‐strands. It is highly acidic as it has only 2 positively charged residues within its 91 amino acids (Ebbinghaus *et al*., 2004).

Strikingly, it has been found – by analysing biopsies obtained from different tumours – that most solid tumours and different types of lymphoma express ED‐B‐containing cFN (Sauer *et al*., 2009; Schliemann *et al*., 2009a, 2009b,2009a, 2009b), as do certain types of leukaemia as well (Padro *et al*., 2000). Several studies have also reported a positive correlation between ED‐B expression levels and tumour grade or aggressiveness (Birchler *et al*., 2003; Castellani *et al*., 2002; Sauer *et al*., 2009).

The rationale behind targeting ED‐B in cancer is that it would allow cancer therapeutics to be targeted specifically at tumour cells, while sparing healthy, off‐target cells. In 1998, Pini *et al*. developed an antibody, coded as L19 during the experiment, that bound ED‐B with a 28‐fold higher affinity compared to a large repertoire of functional antibodies with similar expression and performance (Pini *et al*., 1998). This specific antibody has mutations in residues 31–33 and 50–54 of the heavy chain, and in residues 32 and 50 of the light chain (Pini *et al*., 1998; Tomlinson *et al*., 1995). Different antibody variants that bind ED‐B have since been developed and used, such as immunoglobulin G (IgG) (Mohlmann *et al*., 2011), diabody (Danielli *et al*., 2015; Rekers *et al*., 2018; Zegers *et al*., 2015), mini‐antibody/SIP (small immune protein) (Borsi *et al*., 2002; Steiner and Neri, 2011; Tijink *et al*., 2009) and scFv (Menrad and Menssen, 2005; Ventura *et al*., 2011; Viti *et al*., 1999). IgG antibodies (180 kDa) are preferred if long circulatory half‐lives are needed in blood (List and Neri, 2013; Neri and Sondel, 2016), while diabody, mini‐antibody or scFv (45–130 kDA) demonstrates a faster uptake and improved penetration of the tumour at the targeted region, resulting in a lower blood concentration of L19, which is preferred as this prevents or reduces side effects when coupled to a therapeutic agent. However, importantly, the proper patient needs to be selected for this targeted therapy, emphasizing the need of appropriate biomarkers enabling to assess the tumoral ED‐B expression levels. Several possibilities, noninvasively or for conventional diagnosis, will be discussed.

## 3 Targeting ED‐B for imaging

There are different ways to visualize ED‐B in (tumour) tissue, by using noninvasive (SPECT or PET) or invasive [immunohistochemistry, western blot, ELISA or quantitative PCR (qPCR)] imaging methods, the latter necessitating a biopsy. Based on these results, several L19 antibody variants have been coupled to different radioisotopes for use in imaging (Table 1), which have been tested in clinical trials.

**Table 1:** Clinical trials that evaluate L19 bound to different imaging agents.

Name	Brand name	Phase	Total patients (age range)	Description	Cancer type and stage	Organization drug + (expected) publication	Study identifier
^123^I‐L19	NG	NG	20 (34–79 years)	^123^I‐labelled dimeric scFv(L19) antibody fragment	Brain (*n* = 2), lung (*n* = 16) or colorectal (*n* = 2) cancer	Amersham Pharmacia/Santimaria *et al*. (2003)	800/II/I.27.15/1172
^131^I‐L19	Radretumab	NG	3 (27–73 years)	^131^I‐labelled SIP composed of L19	Lymphoma (*n* = 3)	Philogen S.p.A., GE Healthcare/Sauer *et al*. (2009)	Unknown
^131^I‐L19	Radretumab	I	3 (NG)	^131^I‐labelled SIP composed of L19	Non‐small‐cell lung cancer (*n* = 3)	Philogen S.p.A., GE Healthcare/Erba *et al*. (2012)	NCT01124812
^124^I‐L19 ^131^I‐L19	Radretumab	II	6 (NG)	^124^I‐labelled (PET) vs ^131^I‐labelled (SPECT) SIP composed of L19	Non‐small‐cell lung cancer (*n* = 3), breast cancer (*n* = 3)	Philogen S.p.A., Advanced Center Oncology Macerata, GE Healthcare/Poli *et al*. (2013)	NCT01125085

### 3.1 ED‐B detection using noninvasive imaging approaches

**Fig. 1:**
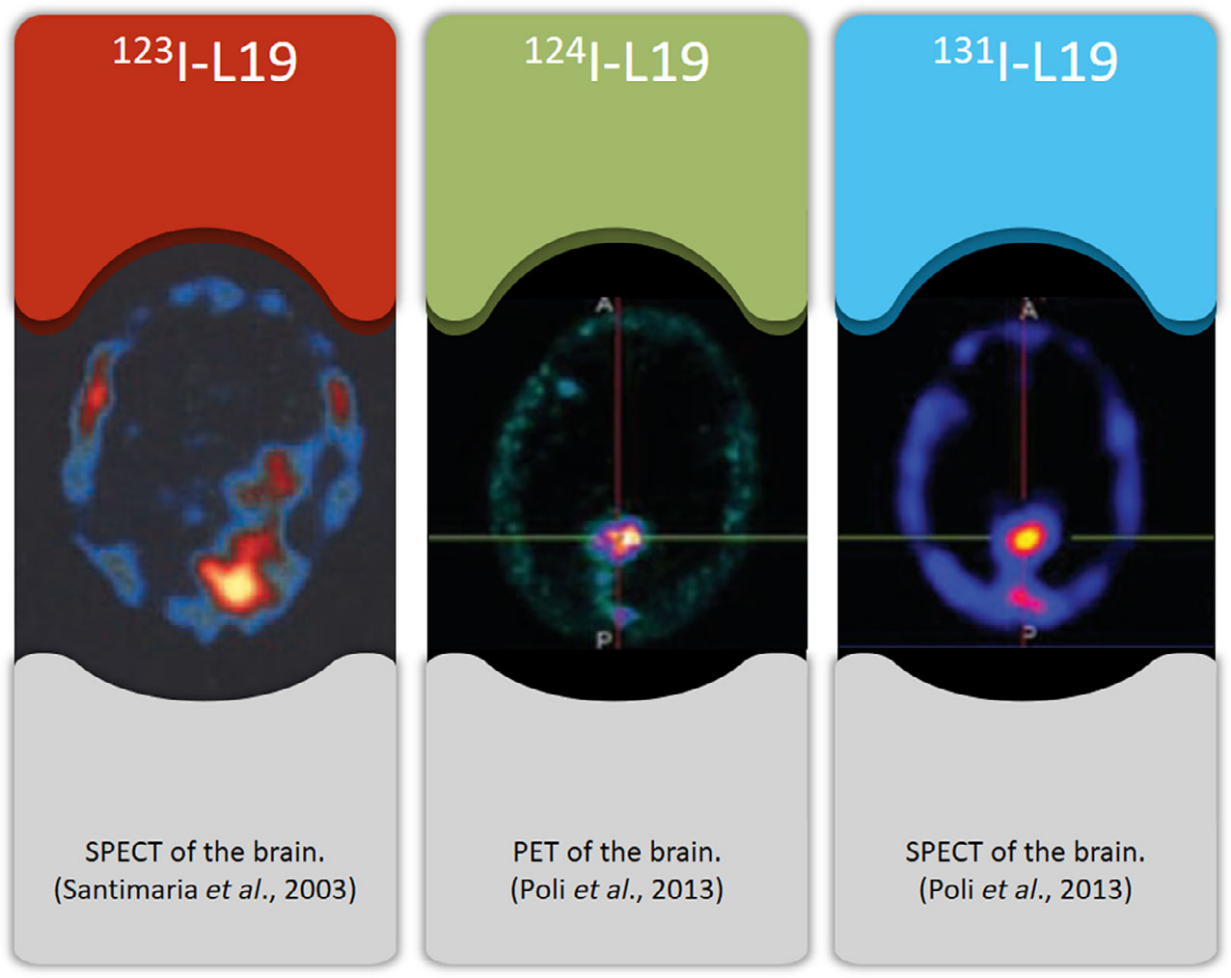
Methods to visualize ED‐B noninvasively in a patients' metastatic brain lesion. Left: SPECT scan where red/orange areas indicate uptake of ^123^I‐L19, middle: PET scan where purple/yellow area indicates uptake of ^124^I‐L19 and right: SPECT scan with uptake of ^131^I‐L19 in the red/yellow area. Images adapted from Santimaria *et al*. (2003) and Poli *et al*. (2013).

Initially, different versions of L19 [IgG, dimeric scFv (scFv)2 and SIP] that target ED‐B were tested for their ED‐B binding capacity before they were coupled to therapeutic drugs. In 2002, Borsi *et al*. showed, in tumour‐bearing mouse models, that the concentration of L19 in the SIP form (L19‐SIP) was seven times higher in tumours than was L19‐(scFv)2, even after 144 h postinjection. Studies carried out using these different forms of L19 have shown that L19‐SIP has a tumour‐to‐blood ratio (TBR) of 70 which means very low concentrations of L19 in blood compared with tumour uptake, L19‐(scFv)2 has a ratio of 10 and L19‐IgG as a ratio of 3 (Borsi *et al*., 2002). In 2003, Santimaria *et al*. performed the first clinical trial of ^123^I‐L19(scFv)2 evaluating its uptake in patients with brain, lung or colorectal cancer. L19‐(scFv)2 was chosen based on its higher TBR compared with that of L19‐IgG, and on its faster clearance from the blood compared with both SIP and IgG, which improves image quality. No adverse effects were observed in response to L19‐(scFv)2 in this safety clinical trial (Santimaria *et al*., 2003). Subsequent to this, the SPECT and PET tracers ^131^I‐L19SIP and ^124^I‐L19SIP were also tested in clinical trials (Fig. 1) (Erba *et al*., 2012; Poli *et al*., 2013; Sauer *et al*., 2009). Sauer *et al*. (2009) analysed the biodistribution, and performed dosimetry evaluations, of ^131^I‐L19SIP [5 mCi (185 MBq)] in relapsed Hodgkin lymphoma patients at different time points after injection. A favourable tumour to bone marrow ratio (TBMR) dosimetry of 14 Gy/1.3 Gy, 22.7 Gy/0.85 Gy and 18 Gy/0.99 Gy for all three patients was observed, with 2/3 patients demonstrating partial remissions. These results support the potential use of ^131^I‐L19SIP as a therapeutic option in the context of radioimmunotherapy (RIT). In 2012, Erba *et al*. confirmed the differential dosimetry of ^131^I‐L19SIP (185 MBq) in tumour to bone marrow with ratios of 10 : 1 in 14/18 treated lymphoma patients using SPECT/CT (Erba *et al*., 2012). SPECT has significant disadvantages compared to the accuracy and quality of immune‐PET on dose delivery prediction (Poli *et al*., 2013). Therefore, Poli *et al*. investigated ^124^I‐L19SIP‐based immune‐PET imaging in patients with metastatic brain lesions from primary non‐small‐cell lung cancer (NSCLC) or breast carcinoma, to test if the accuracy of dosimetric results were better than those achieved using ^131^I‐L19SIP SPECT. The uptake of ^124^I‐L19SIP was highly variable in tumour lesions within the same patient and might explain the short beneficial effects of positive RIT responses (Poli *et al*., 2013).

In 2015, Han *et al*. demonstrated the specific binding of a newly developed peptide, ZD2, to ED‐B (Han *et al*., 2015). ZD2 is currently being tested preclinically as a carrier for imaging purposes, such as for magnetic resonance imaging (MRI) (Ayat *et al*., 2018, 2019; Han *et al*., 2017), SPECT (Ye *et al*., 2017) and MR molecular imaging (MRMI) (Ayat *et al*., 2019). And recently, in 2020, Lui *et al*. developed and tested the ED‐B‐specific cystine‐knot miniprotein, which shows strong tumour uptake in preclinical models by MRMI (Lui *et al*., 2020). Taken together, these data show that L19 can bind with high affinity to the ED‐B located in tumours without causing adverse effects, as demonstrated by the high TBR and TBMR achieved when it is used.

### 3.2 ED‐B detection in tissue biopsies

**Fig. 2:**
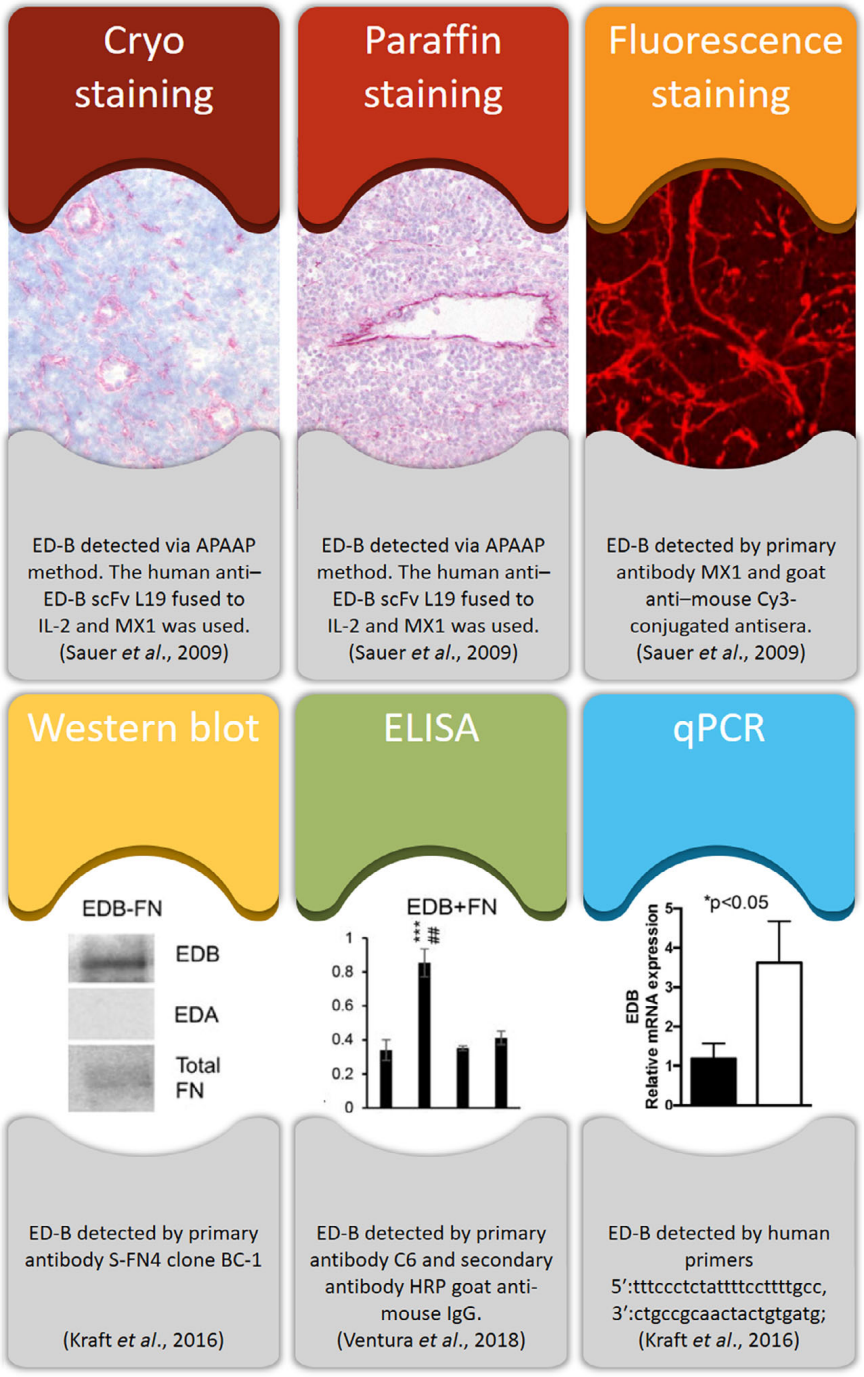
Methods to visualize ED‐B in tissue biopsies. Upper row: biopsies of a lymphoma cancer patient stained for ED‐B. Lower row: cancer patient‐derived material was tested on ED‐B content with western blot and ELISA, and qPCR was used to measure ED‐B in cerebrospinal fluid of patients with bacterial meningitis. Images adapted from Sauer *et al*. (2009) (upper row); Kraft *et al*. (2016) (western blot); Ventura *et al*. (2018) (ELISA); and Kraft *et al*. (2016) (qPCR).

For prognostic evaluation, the visualization and analysis of ED‐B expression levels in blood and (tumour) tissues can be done using a variety of methods, including ELISA, western blot, qPCR and immunohistochemistry (Fig. 2). Using ELISA, the levels of ED‐B can be measured in cell lysates using L19‐SIP as the primary antibody (Kraft *et al*., 2016; Zuberbuhler *et al*., 2009), while RNA analysis can be performed using qPCR with specific primers (Kraft *et al*., 2016; Saw *et al*., 2018; Ventura *et al*., 2018). The most commonly used method for identifying ED‐B in (tumour) tissue sections remains immunohistochemistry, which can be performed on paraffin‐embedded or frozen tumour sections (Danielli *et al*., 2015; Kraft *et al*., 2016; Rekers *et al*., 2018, 2015 Sauer *et al*., 2009; Schliemann *et al*., 2009a, 2009b,2009a, 2009b). Most ED‐B‐targeting antibodies are available online or on request from the manufacturers. For paraffin sections, only the alkaline phosphatase anti‐alkaline phosphatase method combined with the L19 protein is successful (Sauer *et al*., 2009), whereas for fresh‐frozen tissues, several options are available. These include DAB staining with the L19 antibody (Schliemann *et al*., 2009a), fluorescence staining with L19‐SIP as the primary antibody (Menrad and Menssen, 2005; Rekers *et al*., 2015; Zegers *et al*., 2015) or direct fluorescent labelling using L19‐FITC (Puca *et al*., 2019). As most clinically obtained biopsies are paraffin‐embedded, the field would benefit from having more rapid, paraffin‐based methods with which to assess the benefit of L19‐targeted approaches. The paragraphs above highlight possible tools to select patients for L19‐based targeted therapies, from which an overview is given below.

## 4 Targeting ED‐B for treatment

Targeting the tumour microenvironment – specifically, the tumour neovasculature antigen ED‐B – is a more reliable approach compared with targeting tumour cells because tumour cells are known to lose targeted antigens (Neri and Sondel, 2016). L19 and ZD2, the ED‐B specific antibodies, can also be coupled to cytokines and drugs (Table 2). In addition to these antibodies, Saw *et al*. (2018) have developed and preclinically tested a nanoplatform of aptamer‐like peptide (aptide)‐decorated liposomes that bind to ED‐B, which can deliver siRNA to glioblastoma multiforme lesions.

**Table 2:** Clinical trials that evaluate L19 bound to different therapeutic agent.

Name	Brand name	Phase	Total patients (age range)	Description	Cancer type and stage	Organization drug + (expected) publication	Study identifier
L19‐IL‐2	Darleukin	I/II	33 (35–74 years)	IL‐2 bound to L19 diabody	Breast (*n* = 1), thymic (*n* = 1), parotid gland (*n* = 1), peritoneal mesothelioma (*n* = 1), renal cell carcinoma (*n* = 18), colon adenocarcinoma (*n* = 4), rectal adenocarcinoma (*n* = 2), melanoma (*n* = 3), neuroendocrine cervix carcinoma (*n* = 1), biliary tract cell adenocarcinoma (*n* = 1)	Philogen S.p.A./Johannsen *et al*. (2010)	NCT01058538
L19‐IL‐2 + dacarbazine	Darleukin + dacarbazine	II	102 (30–83 years)	IL‐2 bound to L19 diabody + dacarbazine	Melanoma (stage IV) (*n* = 102)	Philogen S.p.A; Eudax S.r.l./Eigentler *et al*. (2011)	NCT01055522
L19‐TNF‐α	Fibromun	I/II	34 (NG)	TNF‐α bound to L19 single‐chain (scFv) human antibody	Colorectal (*n* = 13), thymoma (*n* = 2), cholangiocarcinoma (*n* = 3), parotid adenoid cystic (*n* = 1), gastric adenocarcinoma (*n* = 1), papilla Vater (*n* = 1), unknown primary (*n* = 1)	Philogen S.p.A./Spitaleri *et al*. (2013)	NCT01253837
L19‐TNF‐α + melphalan	Fibromun + melphalan	I	17 (39–85 years)	TNF‐α bound to L19 single‐chain (scFv) human antibody + melphalan	Melanoma [stage III (*n* = 15) or stage IV (*n* = 2)]	Philogen S.p.A.; InnoPharma Inc. Eudax S.r.l./Papadia *et al*. (2013)	NCT01213732
L19‐IL‐2 + gemcitabine	Darleukin + gemcitabine	I	< 28 (NG)	IL‐2 bound to L19 diabody + gemcitabine	Pancreatic cancer (*n* < 28)	Philogen S.p.A./ (Terminated in 2014)	NCT01198522 (lack recruitment)
L19‐IL‐2	Darleukin	II	25 (NG)	Intratumoral application of L19‐IL‐2	Melanoma (stage III) (*n* = 25)	Philogen S.p.A./ Weide *et al*. (2014)	NCT01253096
L19‐IL‐2 + L19‐TNF‐α	Daromun	II	22 (23–79 years)	Intratumoral application of L19‐IL‐2/L19‐TNF‐α	Melanoma [stage III (*n* = 18)/IV (*n* = 4)]	Philogen S.p.A./ Danielli *et al*. (2015)	NCT02076633
L19‐IL‐2 + dacarbazine	Darleukin + dacarbazine	I/II	67 (22–82 years)	IL‐2 bound to L19 diabody + dacarbazine	Melanoma (stage IV) (*n* = 67)	Philogen S.p.A./ Weide *et al*. (2019)	NCT02076646
SABR + L19‐IL‐2	SABR + Darleukin	I	6 (50–75 years)	IL‐2 bound to L19 diabody + SABR	Non‐small‐cell lung cancer, colorectal cancer, renal cell cancer, melanoma	Philogen S.p.A./~ 2020	NCT02086721 (completed)
L19‐TNF‐α + doxorubicin	Fibromun + doxorubicin	I	28 (UN)	TNF‐α bound to L19 single‐chain (scFv) human antibody + doxorubicin	Sarcoma, breast, lung, gynaecological cancer	Philogen S.p.A./~ 2020	NCT02076620 (active, not recruiting)
L19‐IL‐2 + L19‐TNF‐α	Daromun	III	214 (UN)	Intratumoral application of L19‐IL‐2/L19‐TNF‐α	Melanoma (stage III)	Philogen S.p.A./~ 2020	NCT02938299 (recruiting)
L19‐IL‐2 + rituximab	Darleukin + rituximab	I/II	38 (UN)	IL‐2 bound to L19 diabody + rituximab	Diffuse large B‐cell lymphoma	Philogen S.p.A./~ 2020	NCT02957019 (recruiting)
L19‐TNF‐α + doxorubicin	Fibromun + doxorubicin	II	114 (UN)	TNF‐α bound to L19 single‐chain (scFv) human antibody + doxorubicin	Sarcoma (FNLCC grade 2–3)	Philogen S.p.A./~ 2021	NCT03420014 (recruiting)
L19‐TNF‐α	Fibromun	I/II	20 (UN)	TNF‐α bound to L19 single‐chain (scFv) human antibody	Glioma (grade III/IV)	Philogen S.p.A./~ 2021	NCT03779230 (recruiting)
L19‐IL‐2 + L19‐TNF‐α	Daromun	III	248 (UN)	Intratumoral application of L19‐IL‐2/L19‐TNF‐α	Melanoma (stage III)	Philogen S.p.A./~ 2022	NCT03567889 (recruiting)
SABR + L19‐IL‐2 ± aPD(L)1	Darleukin	II	126 (UN)	IL‐2 bound to L19 diabody + SABR ± aPD(L)1	Non‐small‐cell lung cancer (stage IV)	Philogen S.p.A./~ 2023	NCT03705403 (recruiting)

### 4.1 Targeting ED‐B with radioimmunotherapy

In addition to imaging, L19 can be used as a therapy in combination with radiotherapy (L19‐RIT), which emits high doses of radiation on and in the tumour microenvironment. Seven lymphoma patients have received a therapeutic dose of ^123^I‐L19SIP (3.7 GBq) as part of their radiotherapy (RIT) (Erba *et al*., 2012). The duration of the patients' responses after receiving RIT varied between 2 and 7 months, with 29% showing a complete response (no tumour lesions could be found on the PET/CT), 14% showing a partial tumour reduction, 29% showing stable disease (no tumour progression) and 29% showing progressive disease. The beneficial effect of L19‐RIT tumour specificity compared with external beam irradiation is that it can spare healthy tissue from radiation‐induced toxicity. Unfortunately, a second administration of L19‐RIT at a therapeutic dosage did not sufficiently improve long‐term disease control to consider further as a monotherapy (Erba *et al*., 2012; Poli *et al*., 2013). Combining L19‐RIT with chemotherapy, conventional radiotherapy, immunotherapy or other pharmaceutical agents without overlapping toxicities could increase the duration of complete or stable responses (Erba *et al*., 2012; Steiner and Neri, 2011). Indeed, chemotherapy combined with RIT often creates substantial bone marrow toxicity (Steiner and Neri, 2011). However, when L19 is combined with RIT, the resulting TBMR is so low (10 : 1) that bone marrow toxicity is negligible (Erba *et al*., 2012). Combining L19‐RIT with immunotherapy might also have the same beneficial effect as combining external radiation with immunotherapy. The beneficial effect of immunotherapy is that it often creates a memory effect, as is seen in a preclinical study using a combined RT and L19‐IL‐2 therapy (Rekers *et al*., 2018).

### 4.2 Targeting ED‐B using different treatment modalities

#### 4.2.1 L19‐IL‐2

Following antigenic or mitogenic stimulation, T cells typically excrete pro‐inflammatory cytokines, such as IL‐2, to induce the proliferation of T and B lymphocytes, natural killer cells, macrophages and monocytes (Ebbinghaus *et al*., 2004; List and Neri, 2013). Unfortunately, following the injection in patients of therapeutic doses of recombinant IL‐2, substantial toxicities such as capillary leak syndrome and hypotension have often been observed (List and Neri, 2013; Poust *et al*., 2013). Despite these severe adverse effects, IL‐2 remains one of the cytokines able to induce durable remission. Thus, the coupling of IL‐2 to a tumour‐specific carrier is expected to decrease its toxicity to healthy tissue while increasing its therapeutic effects at the tumour site. L19‐IL‐2 is an example of such a specific immunocytokine carrier, in which IL‐2 is coupled to the L19‐scFv fragment. L19‐IL‐2 has been shown in vivo to result into a high uptake by the tumour(s) and low concentration detectable in blood, TBR of 30 : 1, 24 h postadministration (List and Neri, 2013). Additionally, the intravenous injection of L19‐IL‐2 in orthotopic pancreas and hepatocellular carcinoma mice models produced a significant reduction in tumour growth, relative to treatment with IL‐2 at equimolar levels (Menrad and Menssen, 2005). Furthermore, in 2010, L19‐IL‐2 was tested for the first time in a phase 1 safety trial (NCT01058538) in patients with metastatic renal carcinoma. This trial showed that monotherapy L19‐IL‐2 treatment could be safely administered without grade 3 toxicity in a dose‐escalation design, up to 67.5 Mio IU per week for 4–6 weeks. After 2 weeks, the disease stabilized and produced a progression‐free survival maximum of 8 months (Johannsen *et al*., 2010).

Although a reduced tumour burden constitutes a positive outcome, the most desirable primary endpoint for a treatment is a complete response. Given this, combining L19‐IL‐2 with other treatment strategies was a logical next step (Table 2). In 2009, Schliemann *et al*. combined L19‐IL‐2 with rituximab, which resulted in the complete eradication of B‐cell lymphoma in mice (Schliemann *et al*., 2009a), and in the initiation of a phase 1/2 trial in B‐cell lymphoma patients, which is ongoing (NCT02957019). At the same time, another phase 1 trial combined L19‐IL‐2 with dacarbazine (NCT01055522) to treat metastatic melanoma patients. The median progression‐free survival resulting from this combination was 14 months, with one patient showing a complete response for up to 21 months (Eigentler *et al*., 2011). In 2013, a phase 2 trial (NCT02076646) of IL‐19‐IL‐2 combined with dacarbazine was carried out in 67 metastatic melanoma patients. However, the mean progression‐free survival seen in these patients did not differ significantly from that seen in patients treated with dacarbazine alone. When patients were treated with IL‐19‐IL‐2 combined with dacarbazine, a median overall survival of borderline significance was seen at 419 days post‐treatment, relative to 325 days in patients treated with dacarbazine alone. More than half of the patients treated with the combined therapeutic reported a reduction in their symptoms even though the tumour burden did not change significantly (Weide *et al*., 2019).

More recently, systemic L19‐IL‐2 has been combined with local radiotherapy (RT). This combination has produced long‐lasting therapeutic benefit, depending on ED‐B expression levels, RT dose and schedule, and the levels of cytotoxic T cells (Zegers *et al*., 2015) or NK cells (Rekers *et al*., 2015). Also seen were an effect of the nontreated lesions (abscopal effect) and a long‐lasting memory effect (Rekers *et al*., 2018). These positive effects are caused by the absorption of the damage‐associated molecular pattern and antigens, which are released after RT, by dendritic cells, which then present them to T cells in the lymph nodes. The proliferation of the resulting tumour‐specific T cells is then further stimulated by L19‐IL‐2, resulting in the generation of more T cells that can target both irradiated and nonirradiated tumours and initiate a memory effect (Rekers *et al*., 2015). Recently, a phase 1 trial (NCT02086721) was completed in patients with oligometastatic solid tumour that aimed to find the safe dose for stereotactic ablative body radiotherapy (SABR) combined with L19‐IL‐2. This trial showed that it is safe to give 15 Mio IU of L19‐IL‐2 in combination with SABR, which is now being assessed in an international, multicentric, randomized phase 2 trial (ImmunoSABR; NCT03705403).

L19‐IL‐2 can be administered both intravenously and as intratumoral injections as a part of, for example, intralesional immunotherapies (IITs). The first clinical trial (NCT01253096) tested the effect of L19‐IL‐2 as an IIT in stage 3–stage 4 melanoma patients (Weide *et al*., 2014). Following this trial, L19‐IL‐2 combined with L19‐TNF‐α was tested as an IIT in a phase 2 trial (NCT02076633), resulting in a simple and effective method for the local control of inoperable melanoma lesions (Danielli *et al*., 2015). Its beneficial effect was derived from the initiation of a systemic immune response, together with a direct apoptotic response in the injected metastases, which together resulted in the regression of all metastases (Danielli *et al*., 2015). Currently, two phase 3 (NCT02938299 and NCT03567889) IIT trials of L19‐IL‐2 combined with L19‐TNF‐α are ongoing in stage 3 melanoma patients.

#### 4.2.2 L19‐IL‐10

IL‐10 is a potent anti‐inflammatory cytokine that is excreted primarily by monocytes. IL‐10 inhibits cytokines, such as IL‐1, IL‐8 and TNF‐α, and downregulates the expression of MHC2. Its deficiency or total absence can enhance autoimmune pathologies, and IL‐10 has been hypothesized to relieve, or resolve locally, autoimmune disease (Trachsel *et al*., 2007). In 2007, Trachsel *et al*. used the combination of L19‐IL‐10 in arthritic mice based on the high ED‐B expression levels observed in arthritic areas (Kriegsmann *et al*., 2004; Trachsel *et al*., 2007). This combination produced a more prominent therapeutic response on arthritic limbs in this model, compared to mice treated with free IL‐10. Despite these promising results, no clinical follow‐up of L19‐IL‐10 has as yet occurred.

#### 4.2.3 L19‐IL‐12

The pro‐inflammatory IL‐12 is secreted by macrophages, monocytes, neutrophils, dendritic cells and B lymphocytes, after recognizing pathogen antigens. IL‐12 enhances T‐cell differentiation, NK cell activation, interferon gamma (INF‐γ) expression and major histocompatibility complex I (MHCI) processing and presentation (Kumra and Reinhardt, 2016; List and Neri, 2013; Vetizou *et al*., 2015). The injection of recombinant IL‐12 at therapeutic dose levels often initiates substantial toxicities in patients, including anorexia, bone marrow suppression, flu‐like symptoms, hepatotoxicity and even death (Lasek *et al*., 2014; List and Neri, 2013). These adverse effects stimulated preclinical animal studies into the therapeutic potential of IL‐12 coupled to L19 (Halin *et al*., 2002; Ongaro *et al*., 2019). The rationale for this research was, as for IL‐2, to lower the total dose and to decrease the toxicity of the treatment while conserving or increasing its tumour cytotoxic effects. Halin *et al*. (2002) reported that in 129Sv mice bearing F9 teratocarcinoma the total dose of L19‐IL‐12 used was 20‐fold lower compared with IL‐12 alone and that this dose was associated with minimal toxicity and retainment of the same or even better therapeutic response. Gafner *et al*. (2006) and Ongaro *et al*. (2019) also optimized L19‐IL‐12 with protein engineering techniques to obtain a substantial improvement on its performance. Despite these promising results, no clinical trials with L19‐IL‐12 have as yet followed.

#### 4.2.4 L19‐IL‐15

After antigenic or mitogenic stimulation, monocytes and macrophages can secrete the pro‐inflammatory cytokine IL‐15. In preclinical settings, L19‐IL‐15 has a good biodistribution and potent antitumour activity in metastatic F9 and C51 mice (Kaspar *et al*., 2007). However, no (pre)clinical trials of this cytokine are planned.

#### 4.2.5 L19‐TNF‐α

Upon antigenic or mitogenic stimulation, macrophages, monocytes, T and B lymphocytes, and NK cells can secrete tumour necrosis factor alpha (TNF‐α). TNF‐α has pro‐inflammatory activities as it inhibits angiogenesis, increases cytotoxic T‐cell activity and promotes apoptosis. However, it can also promote tumour progression by regulating stromal cell types and by increasing the expression of cytokines, such as CCL2 and IL‐6 (both recruiting myeloid cells), CCL7 and VEGF (both promoting angiogenesis), and TNFR1 and TNFR2 (which are important for regulating tumour‐promoting activities of TNF‐α) (Yao *et al*., 2016). In 2011, L19‐TNF‐α was tested for the first time in a phase 1 trial (NCT01253837) in relapsed metastatic colorectal cancer patients (Spitaleri *et al*., 2013). No data from this trial have been published so far. Soon after this trial, another phase 1 trial (NCT01213732) was initiated in stage 3–stage 4 melanoma patients to investigate a combination of L19‐TNF‐α and melphalan, using the isolated limb perfusion method to reduce systemic toxicity (Papadia *et al*., 2013). A complete response in the treated limb was seen in 50% of patients and was maintained for 12 months. No follow‐up of this trial is as yet planned. Two other clinical trials have just stopped recruiting patients and will soon announce their results on the combined use of L19‐TNF‐α and doxorubicin in sarcoma, breast, lung and gynaecological cancer patients (phase 1; NCT02076620) and in sarcoma only patients (phase 2; NCT03420014). A phase 1 /2 trial (NCT03779230) testing this combination has also just begun to recruit grade 3‐grade 4 glioma patients.

#### 4.2.6 L19‐INF‐γ

Typically, after antigenic or mitogenic stimulation, T cells and NK cells excrete the pro‐inflammatory cytokine INF‐γ, stimulating macrophage activation, MHC1 expression and IL‐1, IL‐6 and TNF‐α secretion (Castro *et al*., 2018). In 1996, Schiller *et al*. tested INF‐γ infusions in a phase 3 trial (no NCT‐code) in metastatic melanoma patients. However, no therapeutic activity was observed (Schiller *et al*., 1996). Ebbinghaus *et al*. (2004) confirmed this finding in a preclinical study with scFvL19‐INF‐γ. Nevertheless, combining L19‐INF‐γ with chemotherapy or with other immunocytokines, such as L19‐IL‐2 or L19‐TNF‐α, could substantially enhance its potency. No clinical follow‐up of L19‐INF‐γ has as yet been planned.

#### 4.2.7 L19‐GM‐CSF

After immune stimulation, the granulocyte–macrophage colony‐stimulating factor (GM‐CSF) is produced by many different cells, such as T cells, macrophages, endothelial cells and even tumour cells. GM‐CSF can act as a hematopoietic growth factor and can stimulate hematopoietic progenitor cells to proliferate (Kaspar *et al*., 2007; Shi *et al*., 2006). GM‐CSF can also act as an immune modulator and stimulates the proliferation of T, NK and B cells. In 2007, Kaspar *et al*. found in a preclinical study that L19‐GM‐CSF had a good biodistribution and potent antitumour activity in metastatic F9 and C51 mice (Kaspar *et al*., 2007). However, (pre)clinical follow‐up remains to occur.

#### 4.2.8 L19‐TF

After vascular damage, TF (tissue factor) interacts with coagulation factor VIIa to form an VIIa‐TF complex that initiates blood coagulation (Rao and Pendurthi, 2005). In 2001, Nilsson *et al*. investigated the safety and efficacy of a truncated form of TF, scFvL19‐tTF, in mice bearing F9, C51 or FE8 tumours to induce a natural occlusion in newly formed tumour vessels (Nilsson *et al*., 2001). No adverse effects were observed, and tumour load was reduced by 30% in all treated mice. However, despite this promising preclinical therapeutic effect, the risk of unwanted occlusions occurring elsewhere in the body is high, especially in patients who are already prone to developing thrombosis or who have underlying pathologies that increase ED‐B expression, such as cardiovascular disease, deficiency in thrombolysis, autoimmune pathology and/or who take medications that enhance blood clotting.

#### 4.2.9 L19‐modified liposomes

Liposomes are being increasingly used as a carrier for drugs, vitamins and cytokines. In 2005, Marty *et al*. used scFvL19‐modified liposomes (L19‐ML) to investigate the safety and specificity of this new delivery system in F9 mice (Marty *et al*., 2002; Marty and Schwendener, 2005). This delivery system works 2–3 times better than free liposomes as it produces higher concentrations in the tumour during the first 2 h after an intravenous injection of L19‐ML filled with ^114m^Indium (Marty and Schwendener, 2005). However, after 6–24 h, no differences in tumour concentration were observed between the two liposome types. The mice were also treated with cytotoxic L19‐ML(2′‐deoxy‐5‐fluorouridylyl‐*N*4‐octadecyl‐1‐β‐d‐arabinofuranosylcytosine), and all showed 62–90% tumour shrinkage on days 5 and 8, compared with free liposome‐treated animals. Despite these very promising results, no clinical follow‐up of L19‐ML (drug or cytokine) has taken place.

Overall, we can conclude that L19 has been successfully coupled to different therapeutic agents, from which some (daromun) has already advanced in phase 3 clinical trials. Others are in different stages of development, from preclinical studies into early clinical testing.

## 5 Conclusion

**Fig. 3:**
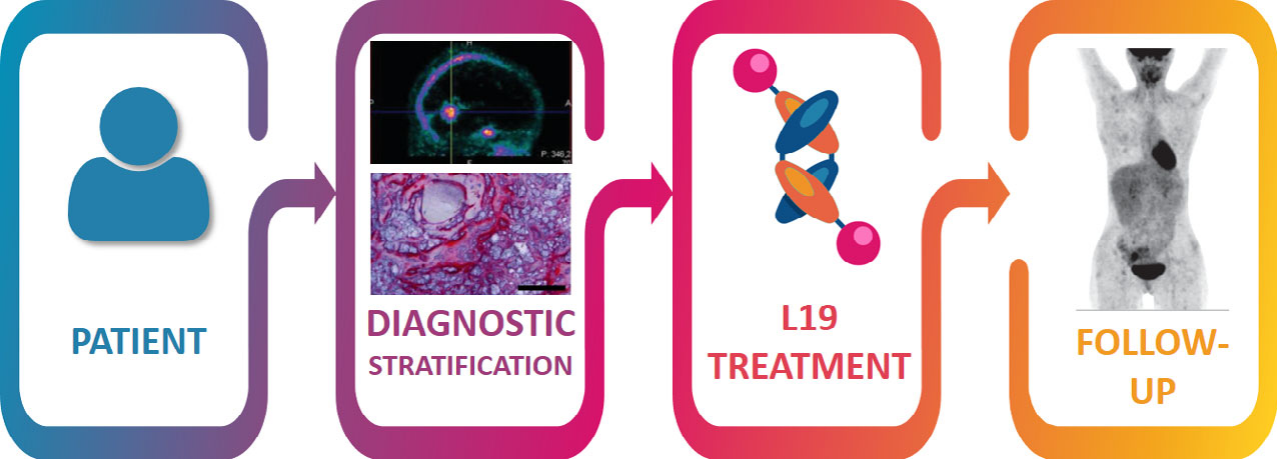
Approach towards clinical implementation of L19 antibody‐based therapeutics. Images adapted from Poli *et al*. (2013), Schliemann *et al*. (2009b) and Steiner and Neri (2011).

Extra domain B is well‐described, preclinically and clinically, and has known applications in imaging and in targeting therapeutics in cancer, as shown by several ongoing phase 2 and phase 3 clinical trials in the context of novel drug development. To further advance the use of ED‐B targeting as a cancer therapeutic and its testing in clinical trials (Fig. 3), we need more experimental studies to be carried out to assess the threshold expression of ED‐B required for L19 treatment modalities to be effective. Furthermore, to enable patients to be properly stratified for ED‐B‐targeted therapy, we need appropriate diagnostic quantification tools to be developed to detect ED‐B, such as noninvasive imaging methods or staining methods that can quickly and easily be used on paraffin‐embedded diagnostic biopsies. Such tools could then also be used to monitor treatment efficacy. Substantial advances have already been made in the targeting of ED‐B for both imaging and therapeutic purposes in preclinical and clinical settings. One therapeutic combination (daromun) is close to being successfully implemented as a novel antibody‐based targeted therapy, particularly for melanoma cancer patients, for which the results are eagerly awaiting for. Also, the combination of conventional treatments such as chemotherapy (dacarbazine) or radiotherapy (SABR) and L19‐based immunotherapies is advancing fast, as evidenced by several phase 2 clinical trials currently ongoing.

## Conflict of interest

PL reports, within the submitted work, a status of PI of the trial ImmunoSABR. PL reports outside the submitted work grants/sponsored research agreements from Varian medical, Oncoradiomics, ptTheragnostic/DNAmito and Health Innovation Ventures. He received an advisor/presenter fee and/or reimbursement of travel costs/external grant writing fee and/or *in kind* manpower contribution from Oncoradiomics, BHV, Merck, Varian, Elekta, ptTheragnostic and Convert pharmaceuticals. PL has shares in the company Oncoradiomics SA, Convert pharmaceuticals SA and the Medical Cloud Company SPRL and is co‐inventor of two issued patents with royalties on radiomics (PCT/NL2014/050248 and PCT/NL2014/050728) licensed to Oncoradiomics and one issue patent on mtDNA (PCT/EP2014/059089) licensed to ptTheragnostic/DNAmito, three nonpatented invention (softwares) licensed to ptTheragnostic/DNAmito, Oncoradiomics and Health Innovation Ventures and three nonissues, nonlicensed patents on Deep Learning‐Radiomics and Lymphocytes Sparing Radiotherapy (N2024482, N2024889 and N2024889).

## Author contributions

RIYL and LJD wrote this manuscript. LJD, PL, AY, JT and RIYL conceived the need of the present review. DM, AMAW and EJVL verified with their expertise literature findings regarding ED‐B. All authors read and approved the final manuscript.
